# Dietary intake of protein and fat of 12- to 36-month-old children in a Dutch Total Diet Study

**DOI:** 10.1007/s00394-021-02653-6

**Published:** 2021-08-24

**Authors:** Annemieke Maria Pustjens, Jacqueline Jozefine Maria Castenmiller, Jan Dirk te Biesebeek, Polly Ester Boon

**Affiliations:** 1 Wageningen Food Safety Research (WFSR), Wageningen University and Research, P.O. box 230, 6700 AE Wageningen, The Netherlands; 2 Netherlands Food and Consumer Product Safety Authority (NVWA), P.O. box 43006, 3540 AA Utrecht, The Netherlands; 3 National Institute for Public Health and the Environment (RIVM), P.O. box 1, 3720 BA Bilthoven, The Netherlands

**Keywords:** Dietary intake, Protein, Fat, Total diet study, The Netherlands, Young children

## Abstract

**Purpose:**

This study attempted gaining insight into the intake of protein and fat of 12- to 36-month-old children in the Netherlands.

**Methods:**

In 2017, a Total Diet Study (TDS) was carried out in the Netherlands including following three age groups: 12–17-, 18–23- and 24- to 36-month-old children. Protein and fat concentrations of 164 composite samples were analysed and combined with the consumption data from the Dutch National Food Consumption Survey 2012–2016 (DNFCS).

**Results:**

Median protein intake of the 12- to 35-month-old Dutch children based on the TDS was 35 g/day with main contributions from the food subgroups “milk and milk-based beverages”, “beef” and “yoghurts and desserts”. Median fat intake was 34 g/day with main contributions from the food subgroups “margarines”, “cheeses” and “milk and milk-based beverages”. For the youngest age group (12- to 18-month-old children), (ready to drink) follow-on formula was one of the main contributors to the fat intake.

**Conclusion:**

Compared to the EFSA reference values, protein intake of the Dutch 12- to 36-month-old children is high, whereas fat intake follows the reference intake. A TDS is a suitable instrument to estimate macronutrient intakes.

**Supplementary Information:**

The online version contains supplementary material available at 10.1007/s00394-021-02653-6.

## Introduction

To estimate the dietary intake of nutrients, food consumption data are combined with food composition data. Food composition data are stored in national food composition databases and are primarily based on chemical analyses of representative samples of foods and beverages. However, when data are missing, they may be borrowed from other countries or data of comparable foods or beverages are used to estimate the intake of nutrients. For example, food composition databases do not always contain information on specific foods specifically aimed at children. In those cases, foods consumed by older age groups are used to estimate the intake of (young) children. Moreover, food composition data are sometimes old and outdated.

Insight into the intake of nutrients can also be obtained by collecting and chemically analysing duplicate portions or by performing a Total Diet Study (TDS). In the case of a TDS, foods and beverages actually consumed by a study population are analysed for chemicals and used to estimate the intake. Foods and beverages included in a TDS should be representative of the consumption pattern of the population group studied. Chemicals are analysed in (pooled) samples of foods and beverages purchased at retail level and prepared as consumed, where applicable. A TDS is, therefore, considered ‘to provide a solid basis for calculating population dietary exposure and assessing potential impact on public health’ [1].

In 2018, food consumption data of the Dutch National Food Consumption Survey (DNFCS) 2012–2016 among persons 1–79 years of age were published [2, 3]. In 2016, food consumption data of the first 2 years of the DNFCS became available [4]. These data were used to set up a TDS covering foods and beverages consumed by children aged 12–35 months in the Netherlands. This age group was selected because there is limited information on the intake of chemicals in this age group. Furthermore, young children consume higher amounts of foods per kg body weight and may thus be exposed to higher concentrations of chemicals per kilogram body weight than adults. The aim of the TDS was to calculate the long-term intake of protein, fat, mycotoxins and heavy metals for this age group.

In this paper, the design of the TDS is described together with the results of the long-term intakes of protein and fat. These results will be compared to European dietary reference values for protein and total fat.

## Materials and methods

### Total diet study (TDS)

The TDS was based on food consumption data of children aged 12–17, 18–23 and 24–35 months, collected during the first 2 years of the Dutch National Food Consumption Survey (DNFCS) of 2012–2016 [4]. Food consumption data of 232 children aged 12 up to and including 35 months were collected via two non-consecutive 24-h dietary recalls in combination with a food diary using GloboDiet. GloboDiet is a computerised interview-based dietary assessment tool for detailed recall of all items a person has eaten and drunk during 24 h. It was developed and is maintained by the International Agency for Research on Cancer in Lyon, France.

For the TDS, the consumed foods and beverages were grouped in 18 food groups and 59 subgroups, and ranked according to their consumed amounts per subgroup for inclusion in the composite samples (Supplementary Table 1). Considering that foods and beverages consumed by young children change with age, the grouping of the consumed products was carried out for the following three age groups: 12–17, 18–23 and 24–35 months.

In total, 1930 foods and beverages were grouped in 164 composite samples. These foods and beverages covered 96–98% of the consumed amounts of all foods and beverages in the three age groups. A small number of foods were not included in the TDS because they were only consumed in a very small amounts or not available on the market (e.g., confectionery typically only available in December). At least 12 products were included in a composite sample. The sample size of 12 per food or beverage was based on an analysis performed for a previous Dutch TDS on mycotoxins [5]. All foods and beverages were bought from supermarkets, market places and specialised shops (such as a bakery or butcher) in one area (Wageningen and surroundings) between August and November 2017, based on market shares. With regard to supermarkets, foods and beverages were bought at six different supermarkets, which covered 79% of the sales via supermarkets in the Netherlands [6]. For example, bananas were bought from six supermarkets, a market place and a greengrocer. Purchase of different flavours of a food or beverage and fresh or preserved (can, glass jar, frozen) foods was based on the consumed amounts as recorded in the DNFCS. Different brands (high, middle, and low price) of industrially prepared foods and beverages were purchased from supermarkets and specialised shops, unless the brand was specifically mentioned in the DNFCS.

Fresh foods and beverages were kept at + 4 °C for a maximum of 3 days before further processing. Non-edible parts, such as skins and peels, were removed. Foods and beverages were prepared as consumed based on the information available in the DNFCS, e.g. cooked, fried, peeled or reconstituted. For the preparation, the instructions on the label were applied or a standard cookbook was used.

The prepared or raw/fresh foods and beverages were homogenised and pooled proportionally to their consumed amounts, based on weight, into composite samples per age group and food subgroup. Six subgroups, namely “apple juice”, “banana”, “beef”, “concentrated fruit juices”, “deep-frying fat” and “mushrooms”, consisted of only one food and thus one composite sample for all three age groups. Liquid composite samples were frozen immediately after pooling. A portion of 500 g of all other (solid) composite samples was freeze-dried. The freeze-dried material was homogenised in a laboratory grinder (Retsch GM200). All composite samples were stored at −20 °C until analysis.

### Analyses

#### Protein analysis

The crude protein content of the composite samples was determined in duplicate using the Kjeldahl principle. Samples (1 ± 0.2 g) were destructed with sulphuric acid in the presence of potassium sulphate and copper sulphate. Protein-nitrogen was converted to ammonium ions. After addition of water and sodium hydroxide, ammonia was distilled and captured in boric acid and titrated with 0.1 M hydrochloric acid using the Gerhardt Vapodest (Gerhardt Analytical Systems, Königswinter, Germany). A nitrogen-to-protein ratio of 6.25 was used to calculate the protein content, except for the composite samples of the subgroup “milk and milk-based products”. For these samples, a nitrogen-to-protein ratio of 6.38 was used according to NEN-EN-ISO 8968-1. Protein was not analysed in the composite samples of the food groups “oils and fats” and “non-alcoholic beverages”, except for the subgroups “margarines” and “other juices”, respectively.

#### Fat analysis

Crude fat content was analysed gravimetrically in duplicate after acid hydrolysis and extraction using petroleum ether. Samples (2.5 ± 0.1 g) were cooked in 3 M hydrochloric acid for 1 h, cooled down and filtered. The washed and dried residue was extracted using petroleum ether according to the Soxhlet principle during at least 6 hours. The petroleum ether was subsequently distilled and the remaining fat-fraction was dried and weighed. Fat was not analysed in the composite samples of the food group “non-alcoholic beverages”.

### Intake calculations using TDS samples

The long-term intake of protein and fat was calculated using the Observed Individual Means (OIM) model as implemented in the calculation tool Monte Carlo Risk Assessment (MCRA) version 8.3 [7]. The daily consumed amount of a food or beverage of a child was multiplied with the concentration in the relevant composite sample. These intakes were summed, resulting in an intake per day per child. To obtain a measure for long-term intake, the intakes per day were averaged over the 2 days recorded in the DNFCS. Using a bootstrap approach, the mean, 50th (median; P50) and 95th (P95) percentiles of intake were calculated [8, 9]. The median of both percentiles and the 95th uncertainty interval around the percentiles are reported. The 95th uncertainty interval quantifies the uncertainty of the intakes due to the sample size of the food consumption database.

Consumed foods not included in the TDS (less than 4% of the total consumed amount) were included by using a concentration based on a combination of composite samples. For example, a sausage roll was disaggregated to its relevant ingredients and corresponding weight percentages using a recipe; these ingredients were then linked to the relevant composite samples.

The intake was calculated per age group and for the total age group of 12- to 36-month-old children. The intake was calculated for the total duration of the DNFCS (2012–2016). The 2012–2016 period includes a larger group of children (*n* = 440 as compared to *n* = 232 for the period 2012–2014) and is more representative due to the inclusion of food consumption data of two more years.

## Results

### Foods and beverages consumed

The food consumption (period 2012–2014) per food group and subgroup for each age group used to design the TDS is described in Table 1 and Supplementary Table 2. “Non-alcoholic beverages” comprised 35–45% of the total intake. Main food groups, apart from “non-alcoholic beverages” that contributed most to the total intake of 12- to 36-month-old children were as follows: “dairy products”, “fruit” and “cereals and cereal-based products”. The food group “dairy products” contained milk and milk-based drinks that contributed to the total intake of fluids. The intake of typical children’s meals (ready-made meals) and follow-on formula reduced with age.

**Table 1:** Mean food intake in the period 2012–2014 per food group and age group, in gram per day

Food group	12–17 months	18–23 months	24–35 months
	Intake, g/day	Intake, g/day	Intake, g/day
Cereals and cereal-based products	138.9	111.7	118.9
Children’s meals	27.1	26.5	4.0
Confectionery	18.7	29.3	30.6
Dairy products	276.0	324.7	321.3
Eggs	3.9	4.4	8.2
Fish and shellfish	3.7	5.8	2.1
Fruit	130.3	163.6	127.1
Follow-on formula	92.9	56.2	35.0
Legumes	2.6	1.8	1.3
Meat	25.9	32.2	35.4
Non-alcoholic beverages	436.5	567.6	648.6
Nuts	1.7	3.7	4.4
Oils and fats	7.3	11.0	7.4
Potatoes	29.4	38.2	35.1
Sauces	4.1	5.9	6.1
Savoury snacks	2.6	6.5	3.9
Soy products	18.8	4.6	10.6
Vegetables	39.8	41.6	54.4
Total intake	1260	1436	1456

The Netherlands Nutrition Centre advises 2-year-old children to eat daily 50–100 g vegetables, 1.5 portion of fruit, 2–3 slices of (brown, wholemeal) bread, 1–2 spoons of wholemeal cereal products or 1–2 potatoes, 1 portion of fish/legumes/meat, 15 g nut paste, 2 portions of dairy products, no cheese, 30 g spreadable and cooking fats, and approximately 1 L of fluid (https://www.voedingscentrum.nl/nl/gezond-eten-met-de-schijf-van-vijf/waarop-zijn-de-hoeveelheden-in-het-dagmenu-gebaseerd/moet-iedereen-dezelfde-hoeveelheden-eten-.aspx). Compared to this advice, the intake of vegetables by the children in the TDS was rather low, around 50 g/day. Other intakes were well in line with the advice of the Netherlands Nutrition Centre.

### Protein and fat intake

#### Protein and fat contents of food groups analysed

Minimum and maximum protein and fat concentrations of the composite samples in each food group are listed in Table 2. For information on the protein and fat concentrations of the food subgroups and per age group, see Supplementary Table 3. The concentration of protein varied from 0 g/kg in the food group “vegetables” to 579 g/kg in “meat”. The concentration of fat varied from 0.32 g/kg in the food group “vegetables” to 1000 g/kg in “oils and fats”. Across samples for different age groups, differences in concentrations were small. Within food groups, some food subgroups contained higher protein and fat concentrations than other foods in the group. For example, the food subgroup “cheeses” had higher protein and fat concentrations than “milk and milk-based beverages” or “yoghurts and desserts”, and mushrooms had higher protein and fat concentrations than other (groups of) vegetables.

**Table 2:** Minimum and maximum concentrations of protein and fat in composite samples per food group

Food group	Concentration in grams per kg fresh weight^a^	
	Protein	Fat
Cereals and cereal-based products	27–150 (18)	6.9–89 (18)
Children’s meals	30–30 (3)	19–20 (3)
Confectionery	22–73 (12)	1.7–330 (12)
Dairy products	22–232 (12)	7.5–326 (12)
Eggs	129–144 (3)	116–132 (3)
Fish and shellfish	143–179 (3)	93–120 (3)
Fruits	2.4–28 (22)	0.5–9.6 (22)
Follow-on formula	16–17 (3)	22–27 (3)
Legumes	55–63 (3)	12–84 (3)
Meat	103–579 (19)	59–296 (19)
Non-alcoholic beverages^b.c^	3.3–3.6 (3)	–
Nuts	215–235 (3)	493–553 (3)
Oils and fats^d^	0.9–1.0 (3)	447–1000 (7)
Potatoes	22–25 (3)	25–55 (3)
Sauces	9.9–12 (3)	45–166 (3)
Savoury snacks	67–93 (3)	181–239 (3)
Soy products	32–42 (3)	19–21 (3)
Vegetables	0–33 (27)	0.32–81 (27)

^a^The number in brackets is the number of composite samples analysed for protein or fat per food group

^b^Only the three composite samples belonging to the subgroup “other juices” were analysed for protein

^c^Composite samples of the food group “non-alcoholic beverages” were not analysed for fat

^d^Only the three composite samples belonging to the subgroup “margarines” were analysed for protein

#### Long-term intake of protein

Table 3 describes the mean, median (P50) and P95 intakes of protein and fat and the three food subgroups that contributed most to the intake of these macronutrients for the different age groups and the total age group. Median intakes of protein ranged from 32.5 to 38.1 g/day and the P95 intake from 51.6 to 67.5 g/day. The median intake of the 12- to 36-month-old children was 35.1 g/day. In all three age groups, and also for the whole age group, the largest contributions to the protein intakes came from “milk and milk-based beverages” (17–20%), followed by “beef” and “yoghurts and desserts” (Table 3).

**Table 3:** Long-term intake of protein and fat of children aged 12–35 months based on concentrations analysed in composite samples and the mean contributions (%) of the three subgroups contributing most to the total intake^1^

Macronutrient	Mean, percentile and contribution	Intake in gram per day per age group based on composite samples and mean contribution (%) of the three main food subgroups to the total intake			
		12–17 months	18–23 months	24–35 months	12–35 months
Protein	Mean	32.6 (30.7–34.0)	36.9 (34.8–39.6)	40.7 (38.8–42.7)	37.5 (36.4–38.9)
	P50	32.5 (29.1–34.0)	34.8 (32.0–37.3)	38.1 (35.9–39.3)	35.1 (34.1–36.6)
	P95	51.6 (47.6–57.4)	59.6 (56.1–74.4)	67.5(61.7–74.0)	62.9 (59.4–67.2)
	Main contributions of food subgroups	Milk and milk-based beverages (20%)	Milk and milk-based beverages (17%)	Milk and milk-based beverages (17%)	Milk and milk-based beverages (18%)
		Beef (9%)	Beef (11%)	Beef (15%)	Beef (13%)
		Yoghurts and desserts (8%)	Yoghurts and desserts (9%)	Yoghurts and desserts (8%)	Yoghurts and desserts (8%)
Fat	Mean	29.1 (27.3–31.6)	34.3 (32.3–37.0)	37.5 (36.1–39.7)	34.4 (33.3–35.4)
	P50	27.6 (25.9–30.9)	32.6 (30.2–34.7)	36.5 (35.0–38.9)	33.6 (31.9–34.5)
	P95	45.0 (39.7–48.2)	54.2 (44.8–59.0)	59.6 (56.9–63.7)	57.0 (52.3–58.8)
	Main contributions of food subgroups	Margarines (12%)	Margarines (12%)	Margarines (14%)	Margarines (13%)
		Milk and milk-based beverages (11%)	Cheeses (9%)	Cheeses (10%)	Cheeses (9%)
		Follow-on formula (8%)	Milk and milk-based beverages (8%)	Milk and milk-based beverages (7%)	Milk and milk-based beverages (9%)

^1^P50: median or 50^th^ percentile; P95: 95^th^ percentile; in brackets are the lower and upper limit of the 95% confidence interval

#### Long-term intake of fat

P50 and P95 intakes of fat ranged from 27.6 to 36.5 and 45.0 to 59.6 g/day, respectively (Table 3). For all age groups the main contributing food subgroup to the fat intake was “margarines”. Other food subgroups that made an important contribution to the fat intake were “milk and milk-based beverages” and “follow-on formula” for the youngest age group, and “cheeses” and “milk and milk-based beverages” for the two oldest age groups (Table 3).

#### Comparing protein and fat intakes with dietary reference values

In Table 4, the protein and fat intakes of the children based on the TDS data are compared with the Population Reference Intake (PRI) for protein and Reference Intake (RI) for fat established by the European Food Safety Authority (EFSA) [10, 11]. The median protein intake of the Dutch 12- to 36-month-old children is high compared to the PRI, whereas the median fat intake follows the AI.

**Table 4:** Population Reference Intake (PRI) for protein, Average Requirement (AR) for energy and Reference Intake (RI) for fat (EFSA, 2017/2019) and protein and fat intakes of Dutch 12- to 36-month-old children based on the TDS^1^

Age (years)	Protein						Energy		Fat		
	PRI (g/kg bw/d)	Reference weight (kg)		PRI (g/d)		TDS, P50 (g/d)	AR, MJ/day		RI: 35–40 E% (g/d)^2^		TDS, P50 (g/d)
		M	F	M	F		M	F	M	F	
1	1.14	10.2	9.5	12	11	32	3.3	3.0	30.6–35.6	27.9–31.8	28
1.5	1.03	11.6	10.9	12	11	35					33
2	0.97	12.7	12.1	12	12	38	4.3	4.0	39.9–45.6	37.2–42.5	36

PRI = Population Reference Intake; TDS = Total Diet Study; AR = Average Requirement; RI = Reference Intake; E% = energy%; M = males; F = females; 1 MJ = 238.83 kcal

^1^TDS: 12- to 18-month-old children are reported here as aged 1 year; 18- to 24-month-old children as aged 1.5 years and 23- to 36-month-old children as aged 2 years. Intakes are taken from Table 3

^2^The RI for fat (35–40 E%) is calculated using the AR for energy and 1 g fat = 9 kcal

## Discussion

Children aged 12–35 months represent an age group that can have a different diet or food pattern compared to older population groups, and often information on food consumption and food composition for this age group is sparse. TDSs, mainly involving adults, have been carried out in various countries, resulting in intake estimates of minerals and trace elements [12–14] and of a range of contaminants such as mycotoxins, heavy metals and environmental or processing contaminants (e.g., [15–20]). Estimates of protein and fat intakes based on a TDS have not (to our knowledge) been reported for children. These intakes can provide valuable information and can serve as a validation of the macronutrient intake estimated using food composition databases. In the present TDS, separate composite samples were prepared for following three age groups: 12–17 months, 18–23 months and 24–35 months. In this way, possible differences in consumed amounts and types of foods and beverages consumed between the age groups were addressed.

In the present TDS, intake data from the period 2012to 2014 were used to design the TDS and intake data are presented for the period 2012–2016. This is justified because the intake of protein and fat in the whole period (2012–2016) did not differ significantly from those for the first two years of the DNFCS; all 2012–2014-intakes were within the 95% confidence interval of the 2012–2016-intakes (data not shown). Also the ratio of girls and boys within the two periods did not differ: 0.92 (111 girls and 121 boys) for the 2012–2014 period and 0.94 (213 girls and 227 boys) for the 2014–2016 period.

The intakes of protein and fat of 1- to 3-year-old boys and girls were recently reported by linking the consumed amounts of foods and beverages to the concentrations of protein and fat listed in the Dutch food composition database (NEVO, https://www.rivm.nl/nederlands-voedingsstoffenbestand/toegang-nevo-gegevens/nevo-online) [3]. Upon request, we received the data for 12- to 36-month-old children. P50 and P95 intakes of protein and fat were 38 and 56, and 38 and 62 g/day, respectively. These figures are somewhat (9–12%) higher than the ones calculated in the TDS, except for the P95 intake of protein, that was 11% lower. For the estimation of the habitual intake distributions using the NEVO database, SPADE (Statistical Program to Assess Dietary Exposure) [21] was used, while in the present TDS the Observed Individual Means (OIM) model was used. The use of different models may partly explain the otherwise small differences in intakes. The main difference between these models is that SPADE corrects for the variation in intake within an individual. Due to this, the intake distribution of SPADE will be less broad, resulting in lower P95 estimates compared to those based on the OIM model. The P50 intake is not affected. The Dutch food composition database NEVO contains nutritional information on approximately 2100 foods, including foods for infants and children. For countries with a less complete food composition database, intake calculations based on a TDS are a good alternative.

A possible disadvantage of a TDS is that by pooling foods into samples for analysis, information about the presence and levels of nutrients and other chemicals may be lost due to dilution [1]. Furthermore, the concentrations of individual foods are often not known. It is, therefore, recommended to combine homogeneous foods in a composite sample. This pooling issue is clearly more important for chemicals that may be present in certain foods and is not relevant for nutrients such as protein and fat that are present in almost all foods consumed. Vin et al. [22] described the relevance of a TDS approach for different types of substances regarding four criteria: (1) the substance has to be present in a significant part of the diet or predominantly present in specific food groups, (2) a robust analytical method has to be available, (3) the dilution impact of pooling and (4) the impact of everyday food preparation methods on the concentration of the substance. We have addressed all four criteria in the present TDS and conclude that a TDS is an appropriate instrument to estimate macronutrient intakes.

The foods and beverages included in our study were sampled from August to November 2017 and may not be representative for all foods on the market on an annual basis [23]. However, the presence of protein and fat in foods does not depend on for example, weather or storage conditions and are considered not to vary between seasons and years. Therefore, the intakes of protein and fat are indicative for the intakes of the age groups studied.

In general, the food pattern of the 12- to 36-month-old children followed the advice of the Netherlands Nutrition Centre; however, the intake of vegetables was too low. The protein intake of the Dutch 12–35 months old children was for all three age groups higher than the Population Reference Intake (PRI) of EFSA, while the fat intake followed the EFSA recommendation (Table 4). A similar result was reported from the French Nutri-Bébé 2013 study assessing intakes of 1035 non-breastfed infants and young children using diaries. The protein intake (median intake of 41.4 g/day) in this French study was more than 4 times the average requirement at the age of 30–35 months (more than 3 times the PRI for protein) and the median fat intake (33.1 g/day) was below the EFSA adequate intake (AI) in more than 90% of the children [24]. Fotorek et al. [25] reported on protein and fat intake of 18-, 24- and 36-month-old children using 3-day dietary records between 2004 and 2013 in the DONALD (Dortmund Nutritional and Anthropometric Longitudinally Designed) study. The protein and fat intakes for the three age groups were 30.0–35.4 and 32.9–39.8 g/day (34 energy percent (*E*%)), respectively. In 2017, Walton et al. [26] reported on the results of the National Pre-School Nutrition Survey, a nationally representative sample of 500 Irish children (1–4 years) using a 4-day weighed food-record. Mean intake of 1- and 2-year-old children of protein was 42.4–42.2 g/day (15–16 *E*%) and of total fat 41.8–31.8 g/day (32–34 *E*%). Goldbohm et al. [27] analysed 2-day food consumption records from children aged 12–23 and 24–35 months attending daycare centres in the Netherlands in 2011–2014. The Dutch food composition database (NEVO, edition 2013) was used to calculate intakes. The children aged 12–23 months had a mean daily intake of 43 ± 9 and 38 ± 10 g/day for the intake of protein and fat, respectively. Corresponding intakes for 24- to 35-month-old children were 45 ± 8 and 42 ± 10 g/day. All these results are comparable to our results: a low intake of total fat and a substantially higher intake of protein than recommended. EFSA [10] also noted the high protein intake among children across Europe.

In conclusion, Dutch 12- to 36-month-old children have a high protein intake (median intake of 35 g/day) and an adequate fat intake (median intake of 34 g/day). A TDS is a suitable instrument to estimate macronutrient intakes.

## Supplementary Information

Below is the link to the electronic supplementary material.

Supplementary file1 (DOCX 49 kb)
