# Supplementary material for: Dietary intake of protein and fat of 12- to 36-month-old children in a Dutch Total Diet Study
Source: Eur J Nutr. 2021 Aug 24;61(1):439–46. doi: 10.1007/s00394-021-02653-6 (PMC8783874; doi:10.1007/s00394-021-02653-6)
Supplement: Supplementary file 1 — Supplementary file1 (DOCX 49 kb) [file 394_2021_2653_MOESM1_ESM.docx]

**SUP Table 1** Food groups and subgroups included in the Total Diet Study and composition of composite samples per age group based on consumption data

| Food group and subgroups (number of foods sampled) | Foods present in composite samples  (type and ratio) | Presence in composite sample (in weight%) per age group (in months) | | |
| --- | --- | --- | --- | --- |
|  |  | 12 - 17 | 18 - 23 | 24 - 35 |
| 1. Cereals and cereal-based products | | | | |
| Bread (n=72) | Corn bread  Ginger bread (regular, wholegrain (5:1))  Multigrain bread  Wheat bread (sliced, rolls (1:1))  White bread (water-based, milk-based, rolls (1:1:1))  Whole grain wheat bread (plain, coated with sunflower kernels (1:1)) | 0  6  11  37  22  24 | 2  3  4  45  14  32 | 0  4  12  37  25  22 |
| Breakfast cereals (n=30) | Cereals  Crunchy sweetened muesli  Oat  Unsweetened muesli | 90  0  7  3 | 96  1  0  2 | 83  6  6  5 |
| Crackers (n=24) | Cracker  Crisp bread  Rusk | 90  0  10 | 65  0  35 | 64  10  26 |
| Pasta (n=12) | Macaroni  Noodles  Spaghetti | 46  9  45 | 43  15  42 | 50  0  50 |
| Porridge (n=24) | Porridge, ready to eat  Rice porridge | 100  0 | 84  16 | 100  0 |
| Rice (n=36) | Polished rice  Rice cracker  Rice drink  Unpolished rice | 62  29  0  8 | 74  15  0  12 | 53  16  11  19 |
| 1. Children’s meals | | | | |
| Children’s meals (n=36) | Meal with pasta  Meal with potato  Meal with rice | 24  67  10 | 78  22  0 | 51  49  0 |
| 1. Confectionery | | | | |
| Biscuits (n=90) | Biscuit  Biscuit with raisins  Spiced biscuit (cookies, sprinkles (1:1))  Sponge cake  Toddler/children’s biscuits  Treacle waffle | 32  0  2  0  66  0 | 35  3  5  6  41  10 | 21  3  13  18  25  20 |
| Cakes (n=28) | Apple pie  Cake  Cheese cake  Croissant  Donut  Fruit pie  Whipped cream pie | 40  30  0  13  0  0  17 | 19  38  0  22  21  0  0 | 25  27  17  18  0  12  0 |
| Candies (n=36) | Aniseed sprinkles  Apple treacle  Fruit-flavoured sprinkles  Soft candy ‘Fruitella’  Soft candy/sweets | 6  67  8  0  19 | 8  47  7  3  35 | 8  23  10  7  52 |
| Chocolates (n=60) | Bars  Chocolate candies  Chocolate sprinkles (dark, milk, mixed (1:1:1))  Flakes (dark, milk, mixed (1:1:1))  Spread (milk, hazelnut (1:1)) | 7  8  4  3  79 | 28  6  19  2  45 | 20  4  20  6  51 |
| 1. Dairy products | | | | |
| Cheeses (n=24) | Cheese, Gouda 48+  Cheese, Gouda 20+  Processed spreadable cheese 48+  Processed spreadable cheese 20+ | 41  0  59  0 | 63  0  26  11 | 73  7  17  2 |
| Creams and ice creams (n=36) | Crème fraiche  Ice cream  Whipped cream | 0  9  91 | 18  82  0 | 0  83  17 |
| Milk and milk-based beverages (n=42) | Buttermilk  Chocolate milk, semi-skimmed  Full-fat milk (pasteurised, sterilised (1:1))  Milk beverage  Semi-skimmed milk (pasteurised, sterilised (1:1)) | 4  0  13  3  81 | 3  0  16  4  77 | 4  8  4  9  75 |
| Yoghurts and desserts (n=90) | Children’s dessert  Custard (full-fat, semi-skimmed (1:1))  Greek yoghurt  Quark, plain  Quark, fruit-flavoured  Sweetened yoghurt (skimmed, semi-skimmed (1:1))  Yoghurt (skimmed, semi-skimmed, full-fat (1:1:1))  Yoghurt drink (pasteurised, sterilised (1:1)) | 7  15  14  0  6  12  12  33 | 3  15  0  5  0  14  22  40 | 6  16  0  4  0  0  25  50 |
| 1. Eggs | | | | |
| Eggs (n=12) | Boiled egg  Fried egg | 40  60 | 85  15 | 42  58 |
| 1. Fish and shellfish | | | | |
| Fish and shellfish (n=60) | Fish stick  Fried haddock  Prawn cracker  Salmon (fresh and frozen (1:1))  White fish (fresh and frozen (1:1)) | 47  23  0  0  31 | 23  7  10  32  29 | 78  0  0  22  0 |
| 1. Fruit | | | | |
| Apple (n=12) | Apple, peeled  Apple, unpeeled | 75  25 | 71  27 | 64  36 |
| Apple sauce (n=24) | Apple sauce, plain  Apple sauce, combined with other fruits | 35  65 | 100  0 | 100  0 |
| Banana^1^ (n=12) | Banana | Combined sample: 100 | | |
| Children’s fruits (n=24) | In glass jar  ‘Knijpfruit’ | 96  4 | 68  32 | 80  20 |
| Citrus fruits (n=24) | Mandarin  Mineola  Orange | 89  11  0 | 86  0  14 | 88  0  12 |
| Dried fruits (n=24) | Date  Other  Raisin | 0  10  90 | 0  0  100 | 15  0  85 |
| Other fruits – 1 (n=24) | Grape  Pear, peeled  Pear, unpeeled | 41  42  17 | 9  91  0 | 41  49  10 |
| Other fruits – 2 (n=66) | Blueberry  Kaki  Kiwi  Mango  Melon (Galia melon, watermelon (1:1))  Nectarine  Peach  Pineapple  Strawberry | 0  0  32  12  22  0  21  6  7 | 4  5  44  8  3  12  12  2  11 | 5  0  36  0  11  3  9  7  29 |
| 1. Follow-on formula | | | | |
| Follow-on formula (reconstituted; n=30) | Formula, low in lactose  Formula, 12-18 months (plain/vanilla-flavoured)  Formula, 24-36 months (plain/vanilla-flavoured) | 0  100  0 | 5  95  0 | 7  0  93 |
| 1. Legumes | | | | |
| Legumes (n=30) | Brown beans in glass jar/can  Hummus  Split peas  White beans in glass jar/can | 69  31  0  0 | 46  5  0  50 | 79  2  19  0 |
| 1. Meat | | | | |
| Beef^1^ (n=12) | Minced beef | Combined sample: 100 | | |
| Chicken (n=24) | Chicken fillet  Chicken schnitzel  Deep-fried breaded chicken | 67  5  28 | 68  0  32 | 81  14  5 |
| Meat on bread (n=36) | Bacon  Chicken fillet  Filet American  Ham  Fried minced meat  Sandwich spread/salad | 0  23  0  63  14  0 | 6  24  0  36  11  22 | 12  28  12  44  4  0 |
| Offal (n=18) | Liver pâté  Liver sausage | 73  27 | 75  25 | 60  40 |
| Pork (n=36) | Bacon  Minced meat, pork and beef  Pork schnitzel | 40  0  60 | 44  56  0 | 28  48  23 |
| Sausages (n=24) | Bratwurst  Cooked sausage  ‘Frikandel’  Smoked sausage | 39  13  34  14 | 19  10  45  26 | 15  17  51  16 |
| Sausages on bread (n=24) | Grilled sausage  Luncheon meat  Salami  Saveloy | 33  54  0  13 | 14  21  4  60 | 0  91  9  0 |
| 1. Non-alcoholic beverages | | | | |
| Apple juice^1^ (n=12) | Apple juice | Combined sample: 100 | | |
| Concentrated fruit juices^1^(n=12) | Concentrated fruit juice | Combined sample: 100 | | |
| Other juices (n=36) | Mixed juice  Nectar  Orange juice | 71  18  11 | 57  34  9 | 51  37  13 |
| Soft drinks (n=36) | Cola  Ice tea  Lemonade  Soft drink containing fruit juice | 0  0  16  84 | 22  11  14  53 | 0  18  9  73 |
| Syrups (n=24) | Other syrup  Rose hip syrup | 89  11 | 100  0 | 90  10 |
| Tea (n=24) | Black tea  Green tea  Herbal tea | 100  0  0 | 88  6  6 | 73  9  18 |
| Water (n=12) | Bottled water  Tap water | 3  97 | 0  100 | 1  99 |
| 1. Nuts | | | | |
| Nuts (n=48) | Mixed nuts  Peanut  Peanut butter  Peanut sauce | 0  0  84  16 | 0  0  96  4 | 13  1  72  14 |
| 1. Oils and fats | | | | |
| Deep-frying fat^1^ (n=12) | Deep-frying fat, vegetable | Combined sample: 100 | | |
| Margarines (n=12) | Low-fat margarine  Margarine | 82  18 | 76  24 | 71  29 |
| Oils (n=36) | Olive oil  Peanut oil  Sunflower oil | 64  0  36 | 37  18  45 | 84  0  16 |
| 1. Potatoes | | | | |
| Potatoes (n=36) | French fries  Potato (floury, waxy (1:1))  Potato, precooked | 13  80  7 | 18  78  5 | 32  46  22 |
| 1. Sauces | | | | |
| Sauces (n=48) | Broth  Dinner sauce, no tomato  Gravy  Mayonnaise-like sauce  Pesto  Soy sauce  Spice mix | 1  56  29  6  0  1  6 | 0  36  32  27  0  1  5 | 3  19  24  42  6  1  5 |
| 1. Savoury snacks | | | | |
| Savoury snacks (n=42) | Bread stick  Corn-based savoury biscuit  Popcorn  Potato crisps  Salty biscuit  Salty puff pastry | 44  12  0  32  0  12 | 16  0  1  71  0  11 | 10  11  15  57  6  0 |
| 1. Soy products | | | | |
| Soy products (n=36) | Cooked soy bean  Soy dessert  Soy milk  Soy yoghurt | 0  9  88  3 | 7  0  54  39 | 0  2  78  20 |
| 1. Vegetables | | | | |
| Brassica vegetables (n=48) | Broccoli  Cauliflower (fresh, frozen (1:1))  Red cabbage (fresh, glass jar/can, with apple (1:1:1))  Sauerkraut  White cabbage | 50  38  12  0  0 | 40  23  28  9  0 | 41  45  3  7  4 |
| Onion and leek (n=24) | Leek  Onion | 25  75 | 32  68 | 43  57 |
| Fruiting vegetables (n=48) | Courgette  Cucumber  Gherkin  Sweet pepper | 8  63  6  23 | 8  71  0  21 | 0  86  8  6 |
| Leafy vegetables (n=54) | Chicory  Endive  Lettuce  Spinach (fresh, frozen, with cream (1:1:1)) | 0  38  14  49 | 19  30  0  51 | 7  8  13  71 |
| Mixed vegetables (n=42) | Mixed vegetables (frozen, glass jar/can (1:1))  Pea and carrot (frozen, glass jar/can (1:1))  Soup vegetables  Vegetables to stir fry | 0  20  16  64 | 20  33  33  14 | 20  16  31  33 |
| Mushrooms^1^ (n=12) | Button mushroom | Combined sample: 100 | | |
| Other vegetables (n=36) | Green bean (fresh, frozen, glass jar/can (2:1:1))  Maize, can  Pea (frozen, glass jar/can (1:1)) | 89  2  9 | 85  6  9 | 88  6  5 |
| Root vegetables (n=24) | Beetroot  Carrot | 0  100 | 13  87 | 0  100 |
| Stem vegetables^2^ (n=12) | Asparagus, glass jar  Celery | 100  0 | 0  100 | -  - |
| Tomatoes and tomato products (n=48) | Canned puree  Ketchup  Tomato (regular, cherry, can (1:1:1))  Tomato sauce in glass jar/can | 45  13  33  9 | 21  8  60  11 | 3  8  83  6 |
| Total: 18 food groups (including 1930 foods), 59 food subgroups, 164 composite samples^3^ | | | | |

^1^ One composite sample was prepared for this subgroup.

^2^ No composite sample for the 24-35 months old age group was analysed for this subgroup, because these children did not consume stem vegetables in the Dutch National Food Consumption Survey (DNFCS) of 2012-2014.

^3^ Food groups (n=18) are numbered and in bold in the left column of the table; food groups are composed of food subgroups (n=59), which are also described in bold but without numbers. For most food subgroups (n=53), three composite samples were prepared (one for each age group). For six food subgroups that contained only one food or beverage, one sample was prepared. Stem vegetables were not consumed in the age group 24-35 months. This resulted in 164 composite samples to be analysed.

**SUP Table 2** Mean food intake in the period 2012-2014, per food subgroup and age group, in gram per day and number of consumers^1^

| **Food subgroup** | **12-17 months** | | **18-23 months** | | **24-35 months** | |
| --- | --- | --- | --- | --- | --- | --- |
|  | **g/day** | **No of consumers** | **g/day** | **No of consumers** | **g/day** | **No of consumers** |
| Bread | 62.1 | 27 | 73.7 | 28 | 78.3 | 69 |
| Breakfast cereals | 10.8 | 24 | 8.0 | 28 | 5.6 | 53 |
| Crackers | 1.1 | 11 | 1.1 | 20 | 1.8 | 44 |
| Pasta | 9.5 | 24 | 8.0 | 30 | 13.6 | 63 |
| Porridge | 52.1 | 13 | 12.8 | 6 | 14.5 | 8 |
| Rice | 3.3 | 21 | 8.2 | 26 | 5.2 | 49 |
| Children's meal | 27.1 | 11 | 26.5 | 6 | 4.0 | 3 |
| Biscuits (dry) | 6.9 | 44 | 7.6 | 42 | 8.1 | 97 |
| Cakes | 4.3 | 6 | 5.7 | 13 | 6.3 | 39 |
| Candies | 4.1 | 35 | 7.7 | 41 | 6.5 | 79 |
| Chocolates | 3.4 | 13 | 8.4 | 31 | 9.7 | 81 |
| Cheeses | 8.6 | 35 | 13.5 | 46 | 11.6 | 83 |
| Creams and ice creams | 0.4 | 4 | 1.9 | 12 | 2.4 | 22 |
| Milk and milk-based beverages | 198.0 | 42 | 191.7 | 48 | 197.9 | 99 |
| Yoghurts and desserts | 68.9 | 40 | 117.6 | 49 | 109.5 | 94 |
| Eggs | 3.9 | 14 | 4.4 | 16 | 8.2 | 45 |
| Fish and shellfish | 3.7 | 8 | 5.8 | 14 | 2.1 | 19 |
| Apple | 16.8 | 21 | 28.4 | 31 | 19.8 | 65 |
| Apple sauce | 9.6 | 8 | 8.2 | 12 | 9.4 | 26 |
| Banana | 41.1 | 30 | 58.7 | 41 | 33.5 | 64 |
| Children's fruit | 15.3 | 12 | 10.8 | 8 | 5.1 | 9 |
| Citrus fruits | 14.6 | 12 | 15.3 | 24 | 11.7 | 40 |
| Dried fruits | 2.2 | 14 | 2.5 | 20 | 1.3 | 23 |
| Other fruits-1 | 21.9 | 13 | 23.5 | 21 | 30.1 | 56 |
| Other fruits-2 | 8.9 | 11 | 16.2 | 22 | 16.2 | 46 |
| Follow-on formula | 92.9 | 16 | 56.2 | 12 | 35.0 | 16 |
| Legumes | 2.6 | 6 | 1.8 | 8 | 1.3 | 9 |
| Beef | 3.8 | 21 | 4.2 | 26 | 4.4 | 63 |
| Chicken | 7.9 | 29 | 6.7 | 21 | 5.2 | 44 |
| Meat on bread | 1.7 | 10 | 2.9 | 22 | 3.6 | 28 |
| Offal | 2.7 | 20 | 1.0 | 13 | 1.5 | 27 |
| Pork | 1.1 | 10 | 2.3 | 16 | 6.3 | 37 |
| Sausages | 4.3 | 7 | 10.9 | 21 | 10.3 | 31 |
| Sausages on bread | 4.4 | 6 | 4.3 | 16 | 4.0 | 31 |
| Apple juice | 12.5 | 5 | 59.5 | 8 | 27.8 | 19 |
| Concentrated fruit juices (‘diksap’) | 12.3 | 13 | 6.2 | 7 | 6.5 | 12 |
| Other juices | 50.2 | 20 | 68.7 | 21 | 62.1 | 48 |
| Soft drinks | 23.5 | 5 | 13.7 | 9 | 33.2 | 24 |
| Syrups | 48.8 | 35 | 74.6 | 49 | 122.6 | 94 |
| Tea | 19.1 | - | 64.7 | - | 51.3 | - |
| Water | 270.1 | 4 | 280.2 | 7 | 345.0 | 19 |
| Nuts (peanut) | 1.7 | 12 | 3.7 | 21 | 4.4 | 47 |
| Margarines | 6.3 | 46 | 9.5 | 56 | 6.5 | 116 |
| Oils | 1.0 | - | 1.6 | 3 | 1.0 | 3 |
| Potatoes | 29.4 | 38 | 38.2 | 48 | 35.1 | 93 |
| Sauces | 4.1 | 14 | 5.9 | 29 | 6.1 | 58 |
| Savoury snacks | 2.6 | 17 | 6.5 | 23 | 3.9 | 37 |
| Soy products | 18.8 | 4 | 4.6 | 1 | 10.6 | 9 |
| Brassica vegetables | 9.4 | 22 | 4.9 | 12 | 6.6 | 26 |
| Onion and leek | 1.1 | 16 | 2.7 | 21 | 2.3 | 43 |
| Fruiting vegetables | 2.3 | 14 | 6.4 | 22 | 11.3 | 52 |
| Leafy vegetables | 3.2 | 5 | 5.9 | 14 | 5.5 | 25 |
| Mixed vegetables | 6.0 | 12 | 3.6 | 11 | 2.5 | 25 |
| Mushrooms | 0.7 | 6 | 0.8 | 6 | 0.5 | 14 |
| Other vegetables | 2.9 | 8 | 3.5 | 7 | 7.4 | 30 |
| Root vegetables | 10.5 | 16 | 5.6 | 12 | 5.4 | 28 |
| Stem vegetables | 0.9 | 1 | 0.9 | 3 | - | - |
| Tomatoes and tomato products | 3.1 | 15 | 7.3 | 25 | 12.7 | 66 |

^1^ Total number of children aged 12-17 months: 54; 18-23 months: 58; 24-35 months: 120.

**SUP Table 3** Protein and fat contents of foods samples per age group, in g/kg

|  |  | **12-17 months** | **18-23 months** | **24-35 months** | **12-17 months** | **18-23 months** | **24-35 months** |
| --- | --- | --- | --- | --- | --- | --- | --- |
|  |  | **PROTEIN** | | | **FAT** | | |
| **Cereals and cereal-based products** | Bread | 150.3 | 146.8 | 145.1 | 38.5 | 34.6 | 36.7 |
|  | Breakfast cereals | 108.8 | 107.0 | 106.6 | 38.1 | 37.5 | 45.4 |
|  | Crackers | 118.6 | 125.2 | 123.2 | 88.9 | 84.5 | 81.9 |
|  | Pasta | 52.6 | 60.0 | 50.8 | 8.3 | 8.7 | 8.8 |
|  | Porridge | 27.5 | 27.1 | 27.5 | 20.9 | 21.6 | 20.6 |
|  | Rice | 44.2 | 36.2 | 35.3 | 8.5 | 6.9 | 8.3 |
| **Children's meals** | Children's meals | 30.3 | 29.9 | 30.5 | 19.1 | 18.7 | 20.2 |
| **Confectionery** | Biscuits | 73.2 | 70.7 | 65.8 | 142.7 | 144.1 | 136.5 |
|  | Cakes | 47.2 | 60.9 | 51.7 | 155.4 | 207.4 | 159.5 |
|  | Candies | 24.4 | 21.8 | 45.2 | 1.7 | 3.3 | 5.0 |
|  | Chocolates | 54.2 | 58.7 | 58.3 | 330.1 | 302.3 | 295.7 |
| **Dairy products** | Cheeses | 179.9 | 210.3 | 231.6 | 252.0 | 262.8 | 286.6 |
|  | Creams and ice creams | 21.7 | 28.1 | 27.8 | 326.0 | 148.3 | 147.7 |
|  | Milk and milk-based beverages | 33.5 | 34.2 | 35.7 | 17.1 | 16.1 | 14.4 |
|  | Yoghurts and desserts | 31.9 | 29.7 | 28.4 | 23.5 | 7.5 | 9.9 |
| **Eggs** | Eggs | 144.1 | 129.5 | 141.0 | 132.3 | 116.3 | 118.9 |
| **Fish and shellfish** | Fish and shellfish | 162.9 | 178.6 | 142.6 | 93.3 | 115.0 | 120.4 |
| **Fruit** | Apple | 2.4 | 2.4 | 2.4 | 0.6 | 0.7 | 0.7 |
|  | Apple sauce | 2.9 | 2.5 | 2.5 | 0.6 | 0.6 | 0.5 |
|  | Banana | 10.8 | 10.8 | 10.8 | 0.7 | 0.7 | 0.7 |
|  | Children's fruits | 5.0 | 5.1 | 5.0 | 3.1 | 2.5 | 2.6 |
|  | Citrus fruits | 8.5 | 8.6 | 8.6 | 1.5 | 1.7 | 1.4 |
|  | Dried fruits | 27.2 | 27.9 | 27.5 | 9.6 | 8.2 | 6.9 |
|  | Other fruits - 1 | 7.4 | 7.2 | 7.6 | 2.2 | 2.6 | 2.6 |
|  | Other fruits - 2 | 4.1 | 2.9 | 3.9 | 0.7 | 0.5 | 0.7 |
| **Follow-on formula** | Follow-on formula | 15.8 | 16.7 | 16.7 | 26.9 | 26.9 | 22.0 |
| **Legumes** | Legumes | 63.5 | 55.5 | 62.6 | 84.3 | 22.1 | 12.4 |
| **Meat** | Beef | 578.7 | 578.7 | 578.7 | 162.0 | 162.0 | 162.0 |
|  | Chicken | 298.6 | 290.3 | 309.2 | 82.8 | 82.6 | 59.3 |
|  | Meat on bread | 193.2 | 174.8 | 185.5 | 72.1 | 129.7 | 96.5 |
|  | Offal | 103.3 | 105.7 | 116.6 | 281.4 | 280.5 | 262.5 |
|  | Pork | 278.7 | 307.7 | 469.9 | 165.9 | 245.4 | 206.2 |
|  | Sausages | 168.1 | 156.2 | 147.8 | 206.6 | 200.3 | 188.6 |
|  | Sausages on bread | 153.9 | 189.8 | 131.8 | 258.0 | 296.3 | 258.2 |
| **Non-alcoholic beverages** | Apple juice |  |  |  |  |  |  |
|  | Concentrated fruit juices (‘diksap’) |  |  |  |  |  |  |
|  | Other juices | 3.6 | 3.3 | 3.6 |  |  |  |
|  | Soft drinks |  |  |  |  |  |  |
|  | Syrups |  |  |  |  |  |  |
|  | Tea |  |  |  |  |  |  |
|  | Water |  |  |  |  |  |  |
| **Nuts** | Nuts | 214.7 | 234.8 | 217.0 | 500.0 | 552.8 | 493.4 |
| **Oils and fats** | Deep-frying fat |  |  |  | 998.6 | 998.6 | 998.6 |
|  | Margarines | 0.9 | 0.9 | 1.0 | 447.3 | 463.0 | 488.7 |
|  | Oils |  |  |  | 995.1 | 999.8 | 994.5 |
| **Potatoes** | Potatoes | 21.7 | 22.9 | 25.3 | 25.2 | 34.9 | 55.4 |
| **Sauces** | Sauces | 11.8 | 9.9 | 10.7 | 44.7 | 101.3 | 165.7 |
| **Savoury snacks** | Salty biscuits | 93.3 | 69.6 | 66.8 | 180.9 | 239.2 | 224.6 |
| **Soy products** | Soy products | 32.2 | 41.7 | 34.5 | 19.1 | 20.9 | 19.9 |
| **Vegetables** | Brassica vegetables | 17.8 | 16.2 | 17.2 | 0.8 | 1.7 | 3.1 |
|  | Onion and leek | 14.2 | 14.6 | 13.5 | 35.2 | 32.5 | 24.0 |
|  | Fruiting vegetables | 8.4 | 7.6 | 0.0 | 11.0 | 5.3 | 2.7 |
|  | Leafy vegetables | 18.1 | 19.1 | 18.7 | 2.9 | 24.2 | 16.7 |
|  | Mixed vegetables | 16.0 | 15.8 | 14.3 | 9.3 | 4.7 | 6.8 |
|  | Mushrooms | 32.8 | 32.8 | 32.8 | 80.6 | 80.6 | 80.6 |
|  | Other vegetables | 20.3 | 20.9 | 19.3 | 2.8 | 3.6 | 3.1 |
|  | Root vegetables | 6.6 | 6.8 | 6.3 | 1.1 | 1.8 | 0.9 |
|  | Stem vegetables | 13.6 | 5.0 | 0.0 | 1.8 | 0.3 | 0.0 |
|  | Tomatoes and tomato products | 26.6 | 16.7 | 11.4 | 12.4 | 12.2 | 6.3 |
